# Supplementary figures and images for: Appropriate glycemic management protects the germline but not the uterine environment in hyperglycemia
Source: EMBO Rep. 2024 Mar 15;25(4):11. doi: 10.1038/s44319-024-00097-7 (PMC11014859; doi:10.1038/s44319-024-00097-7)

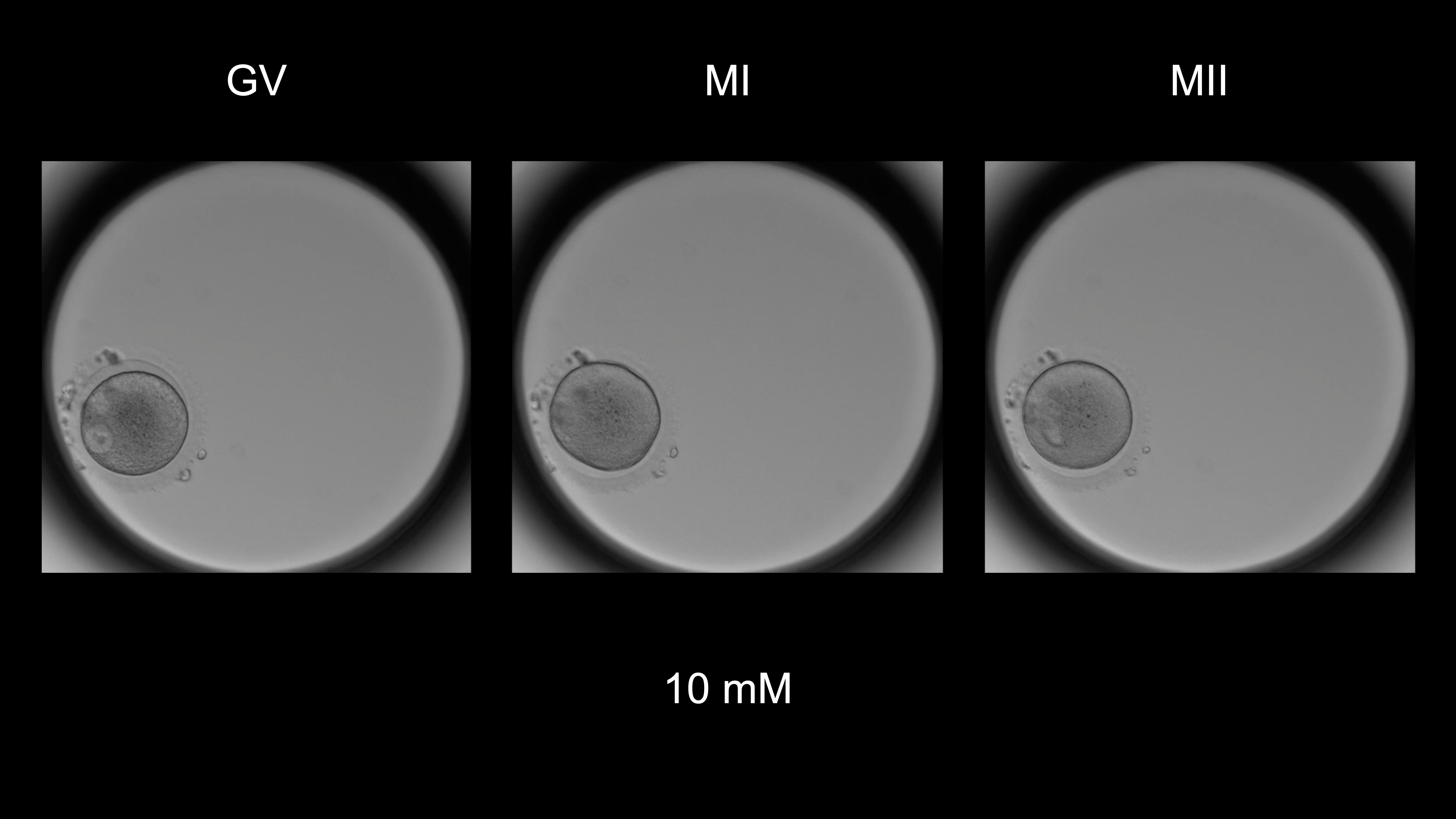

Supplement: Supplementary file 4 — Source Data Fig. 3 [file 44319_2024_97_MOESM4_ESM.zip › Figure 3/3A/10mM_GV_HQ.png]

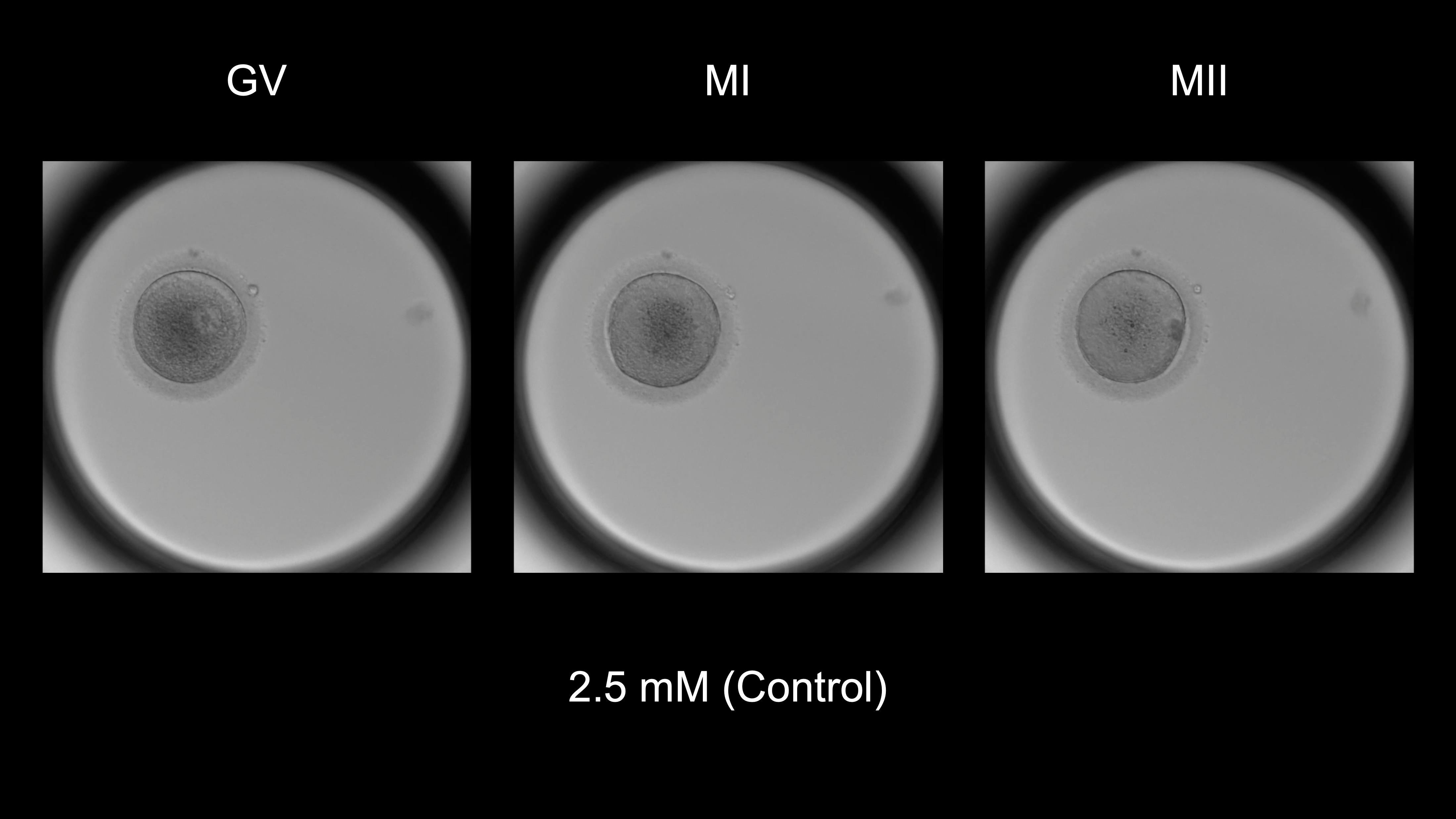

Supplement: Supplementary file 4 — Source Data Fig. 3 [file 44319_2024_97_MOESM4_ESM.zip › Figure 3/3A/Control_GV_HQ.png]

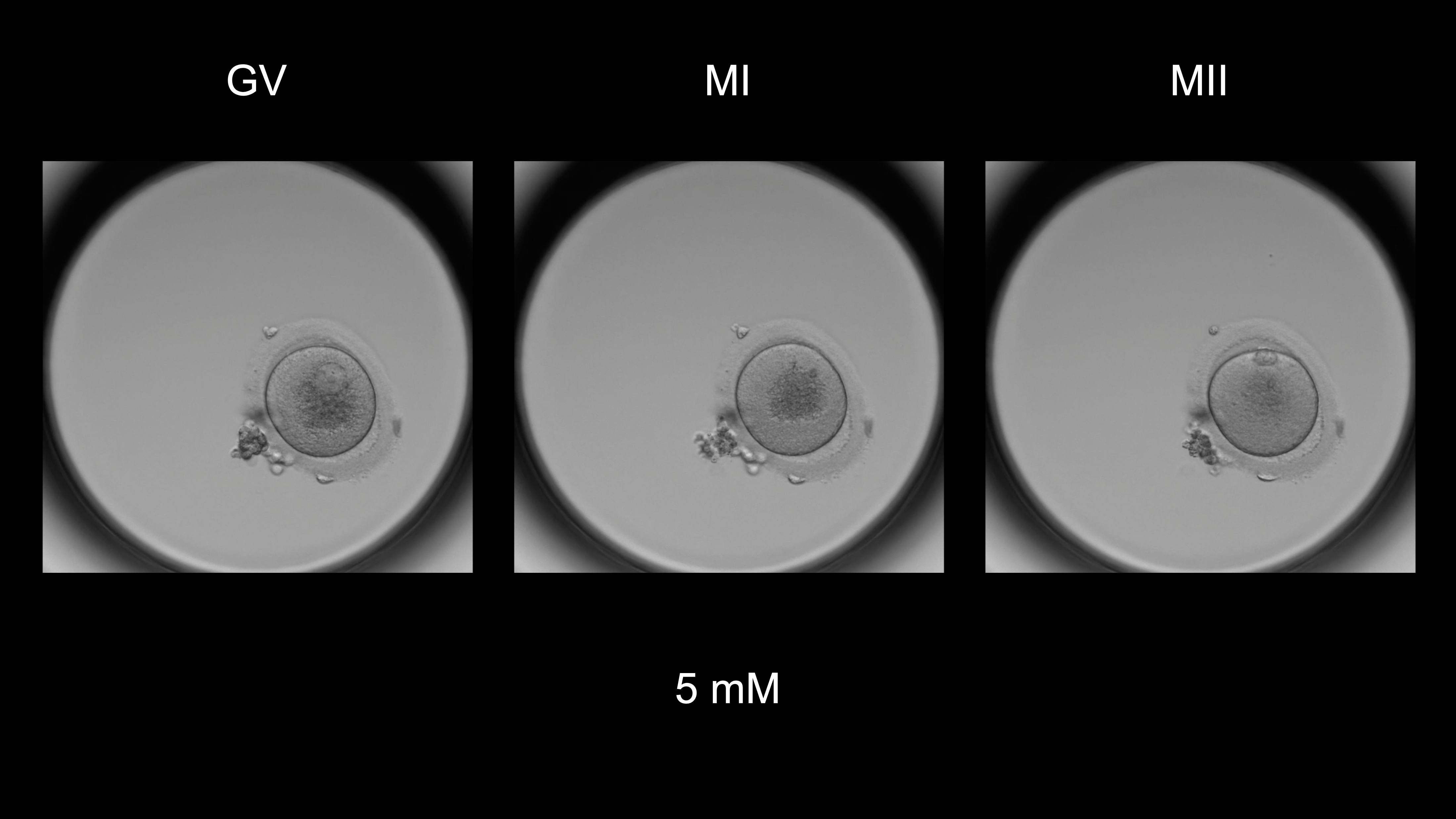

Supplement: Supplementary file 4 — Source Data Fig. 3 [file 44319_2024_97_MOESM4_ESM.zip › Figure 3/3A/5mM_GV_HQ.png]

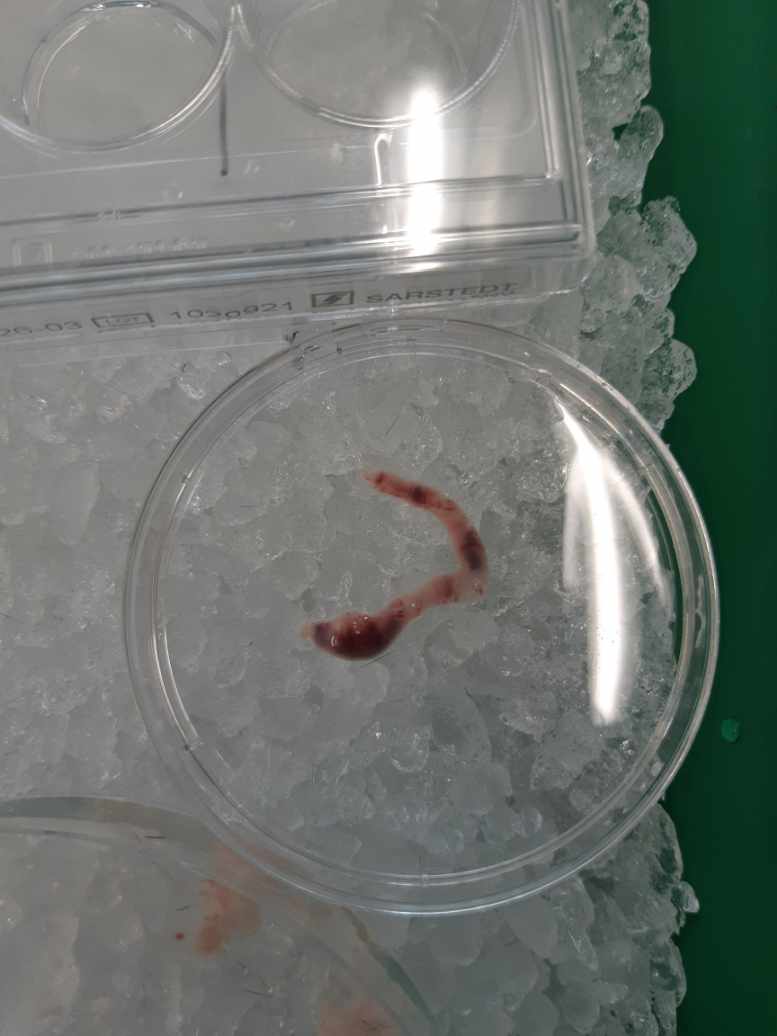

Supplement: Supplementary file 5 — Source Data Fig. 4 [file 44319_2024_97_MOESM5_ESM.zip › Figure 4/4E/STZ uterus.jpg]

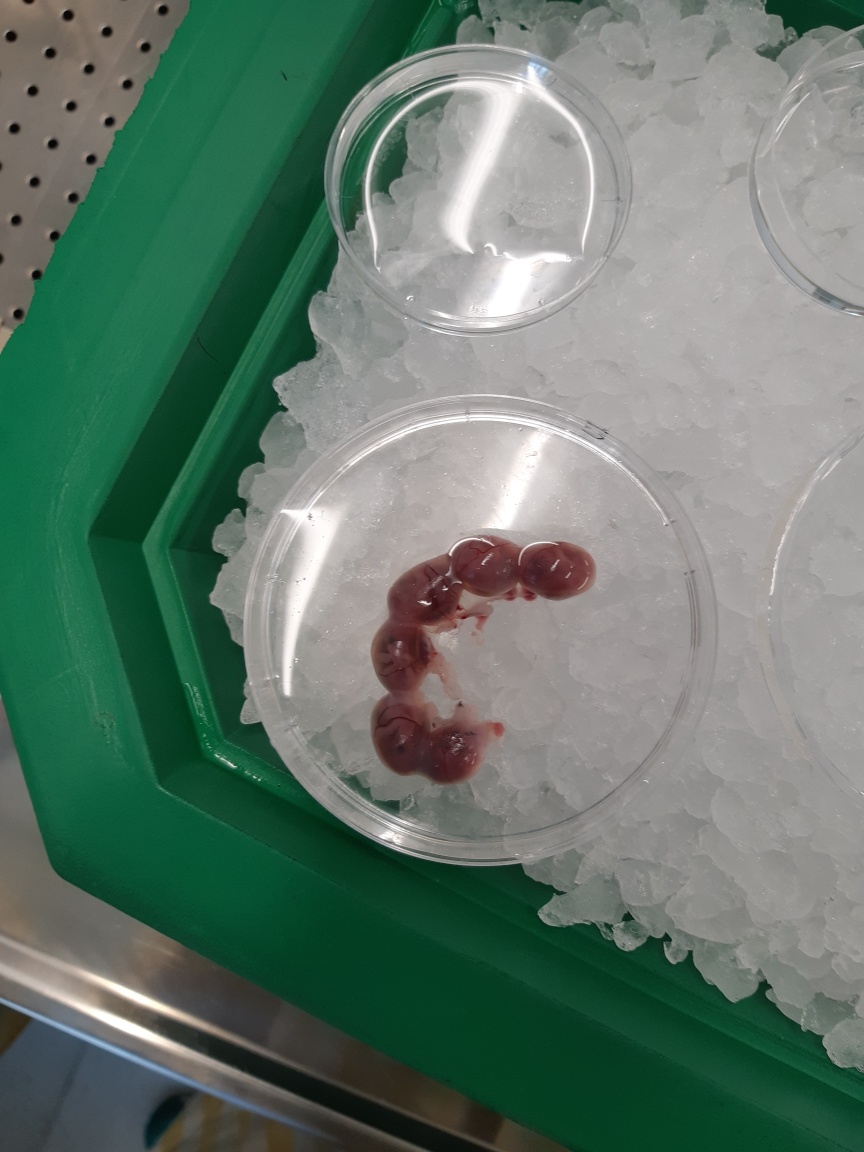

Supplement: Supplementary file 5 — Source Data Fig. 4 [file 44319_2024_97_MOESM5_ESM.zip › Figure 4/4E/ctrl uterus.jpg]

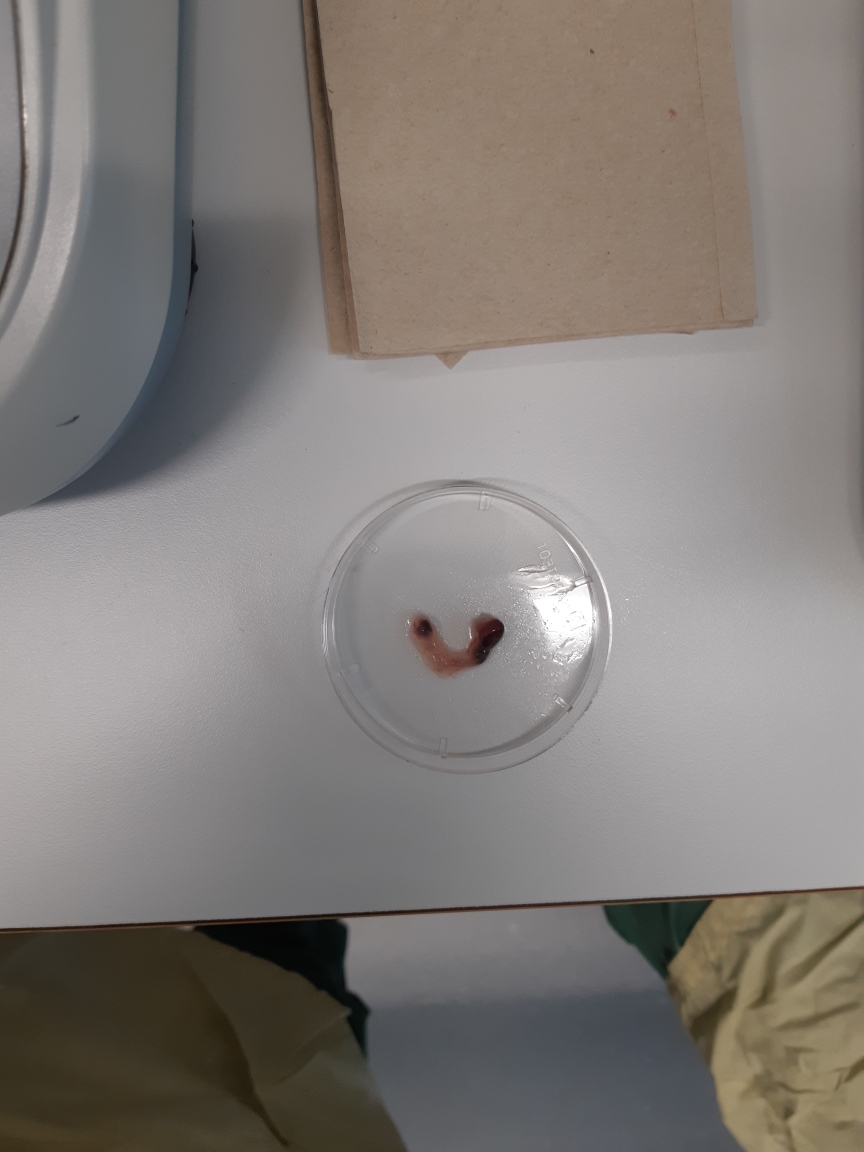

Supplement: Supplementary file 5 — Source Data Fig. 4 [file 44319_2024_97_MOESM5_ESM.zip › Figure 4/4E/STZ uterus2.jpg]

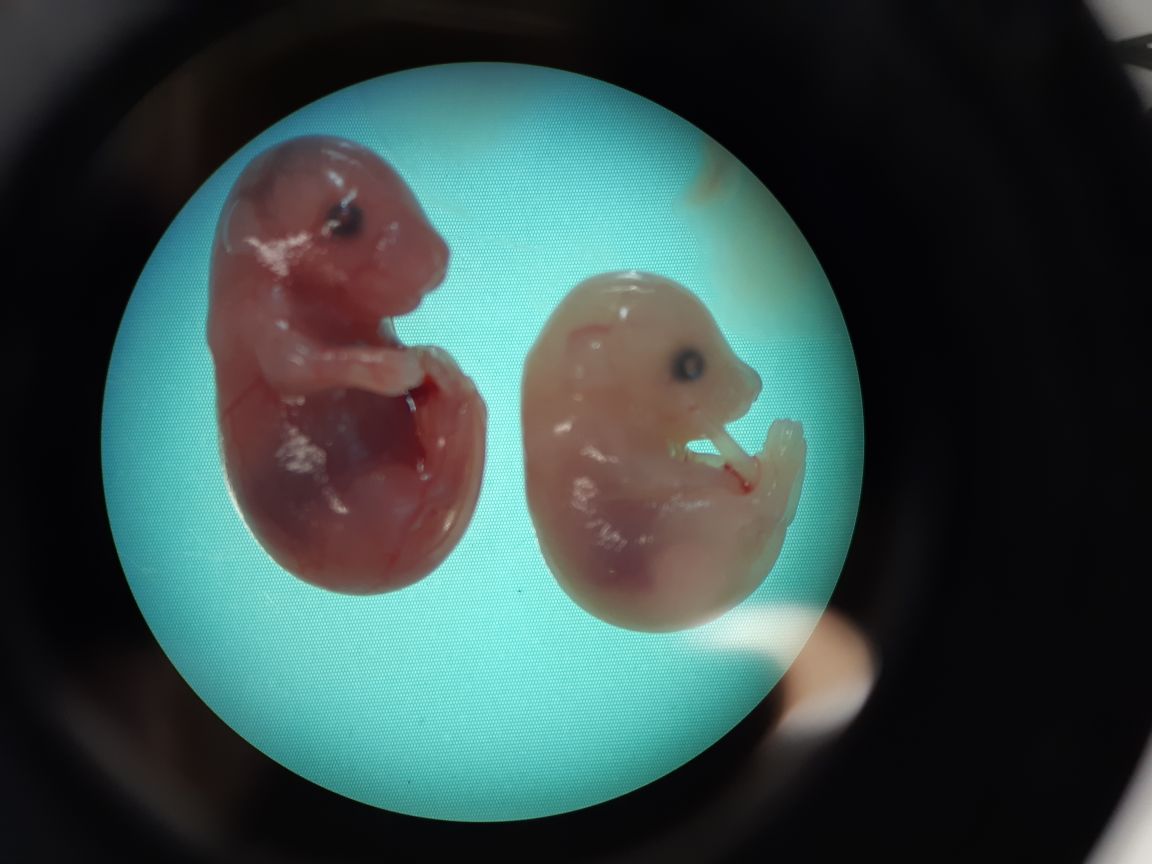

Supplement: Supplementary file 5 — Source Data Fig. 4 [file 44319_2024_97_MOESM5_ESM.zip › Figure 4/4B/embryos.jpg]

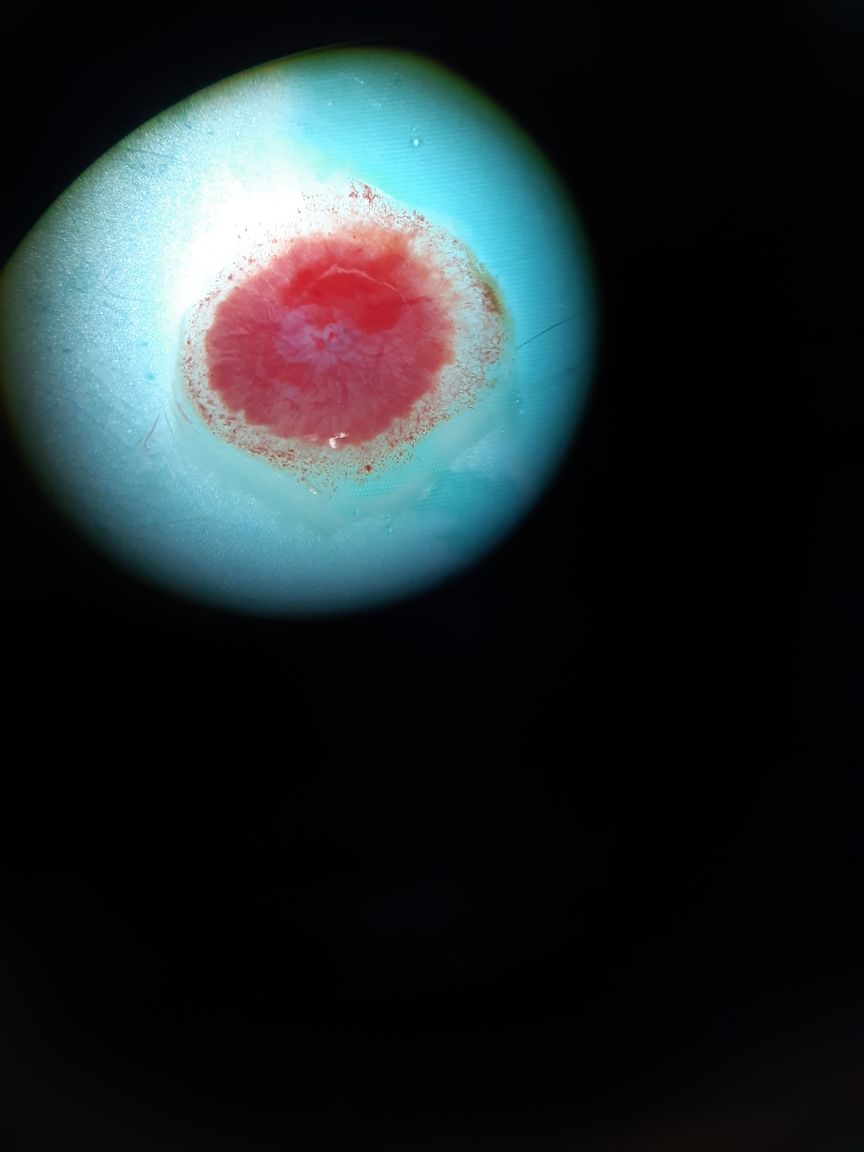

Supplement: Supplementary file 5 — Source Data Fig. 4 [file 44319_2024_97_MOESM5_ESM.zip › Figure 4/4G/ctrl placenta.jpg]

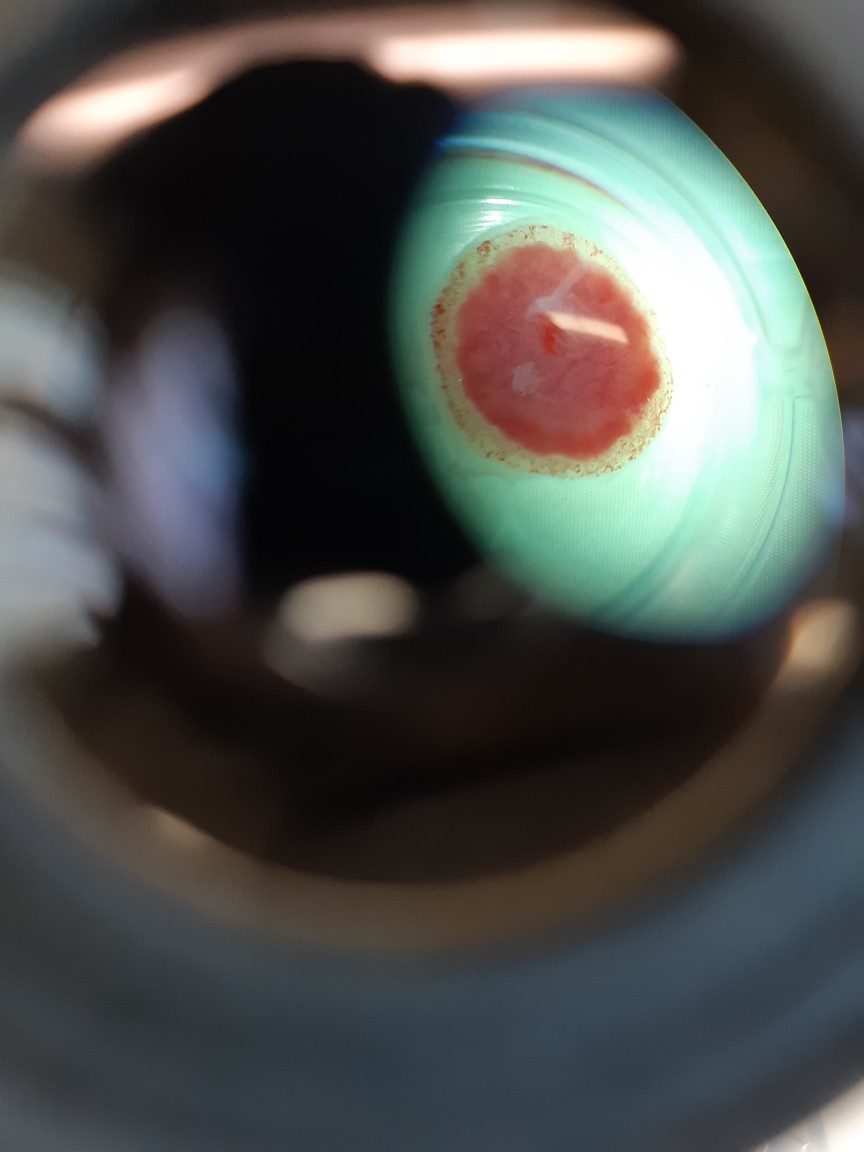

Supplement: Supplementary file 5 — Source Data Fig. 4 [file 44319_2024_97_MOESM5_ESM.zip › Figure 4/4G/STZ placenta.jpg]

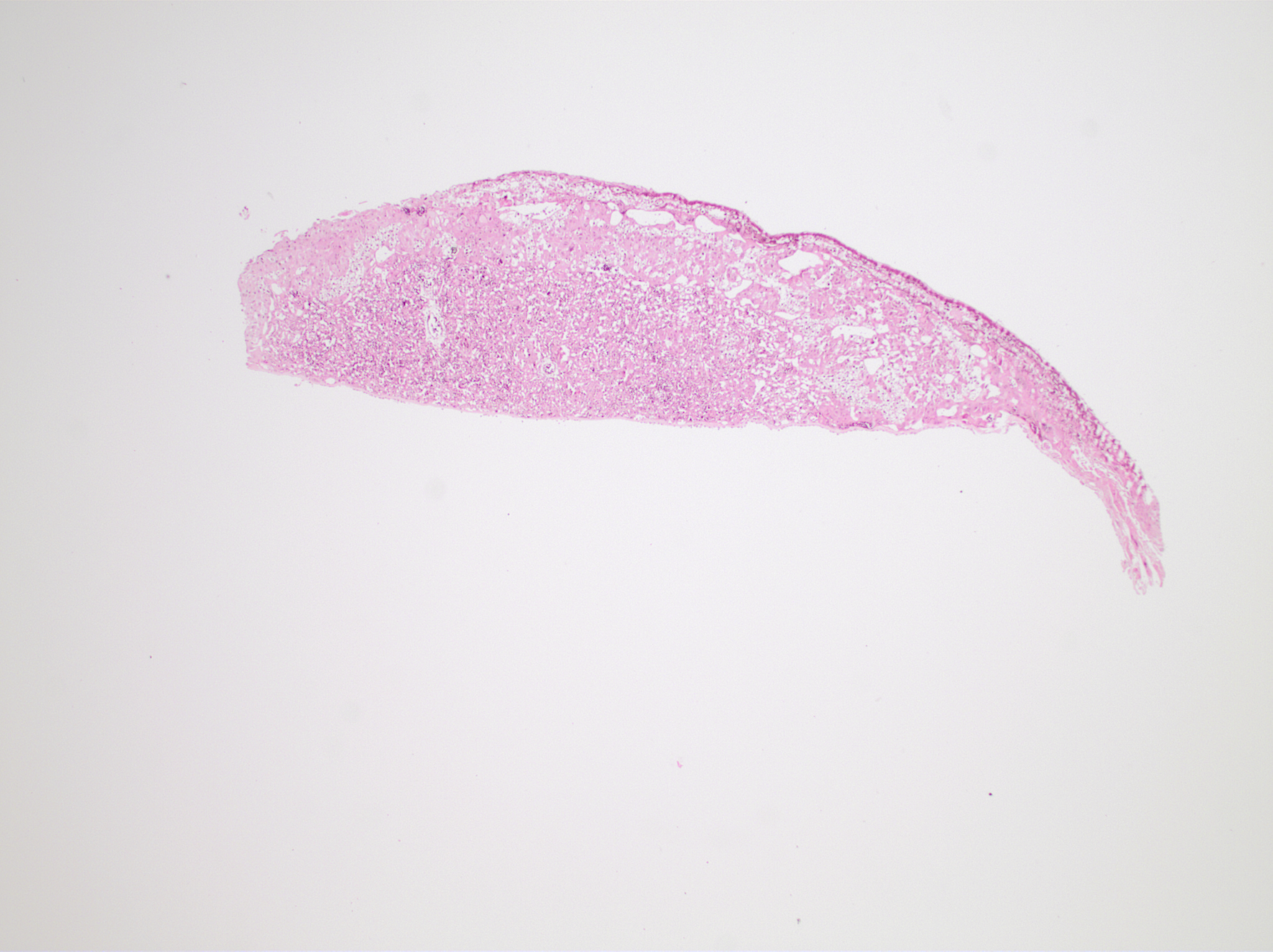

Supplement: Supplementary file 6 — Source Data Fig. 5 [file 44319_2024_97_MOESM6_ESM.zip › Figure 5/5A/Representative_H&E_STZ.tif]

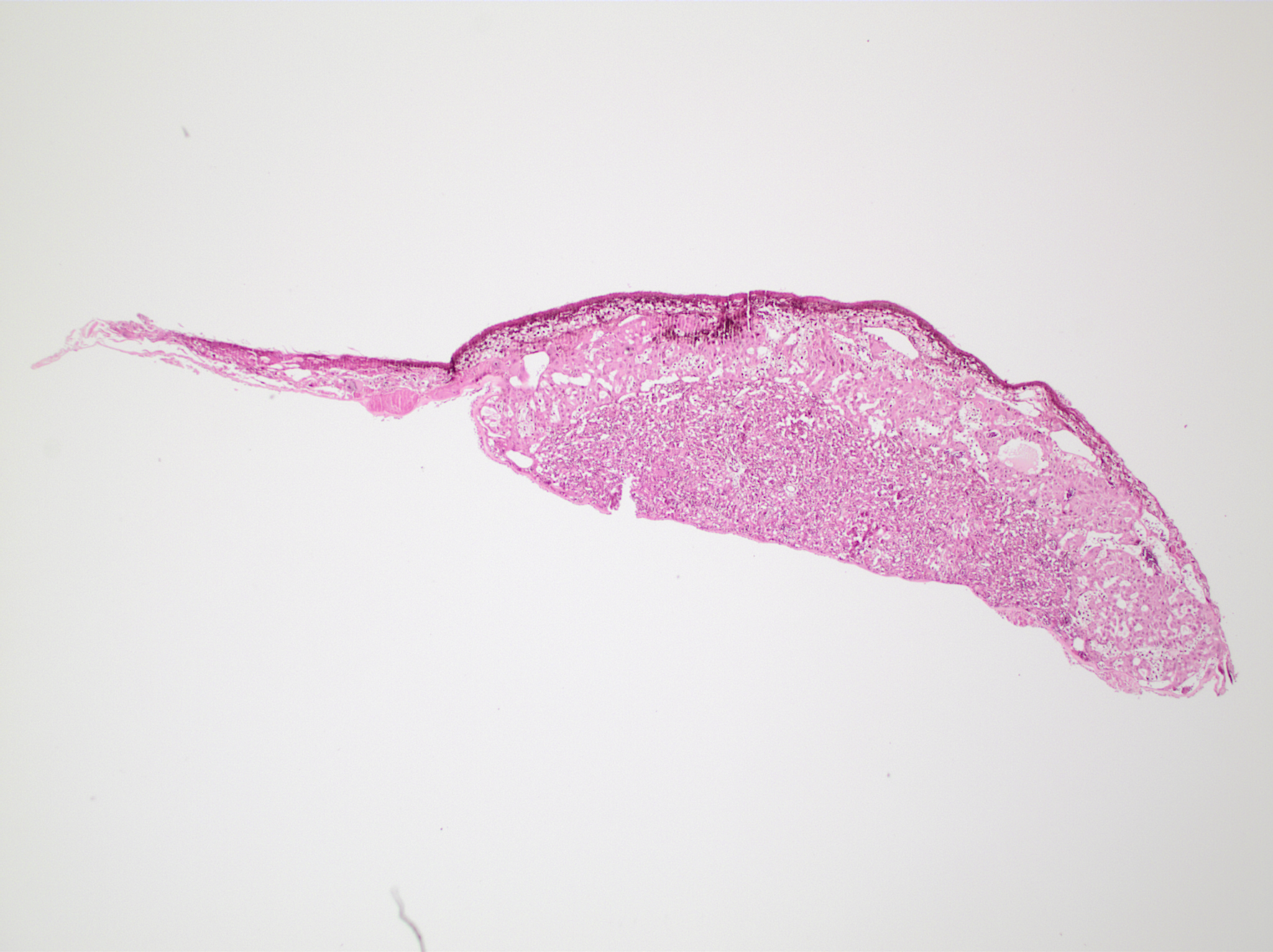

Supplement: Supplementary file 6 — Source Data Fig. 5 [file 44319_2024_97_MOESM6_ESM.zip › Figure 5/5A/Representative_H&E_Control.tif]

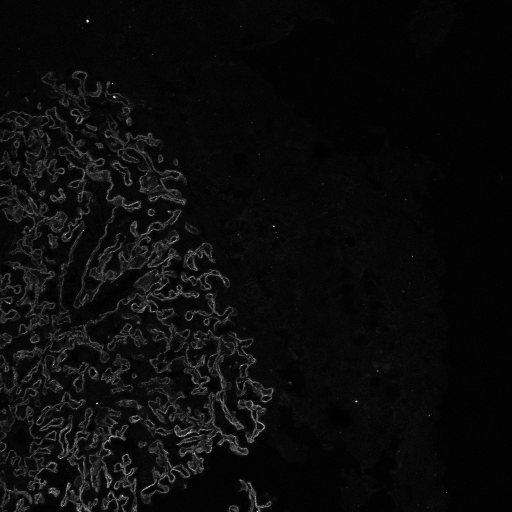

Supplement: Supplementary file 7 — Source Data Fig. 6 [file 44319_2024_97_MOESM7_ESM.zip › Figure 6/6A/Representative_MCT4andCD31_Control.tif]

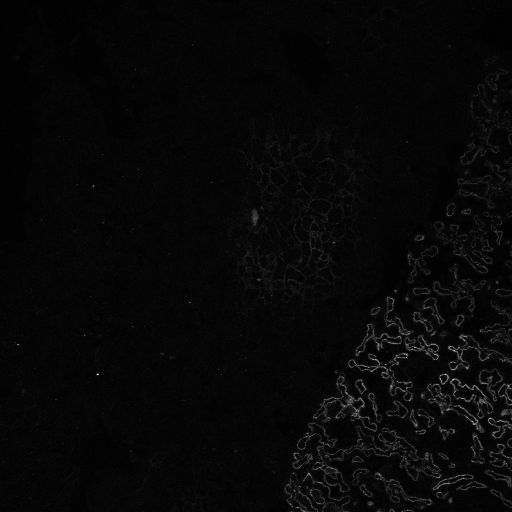

Supplement: Supplementary file 7 — Source Data Fig. 6 [file 44319_2024_97_MOESM7_ESM.zip › Figure 6/6A/Representative_MCT4andCD31_STZ.tif]

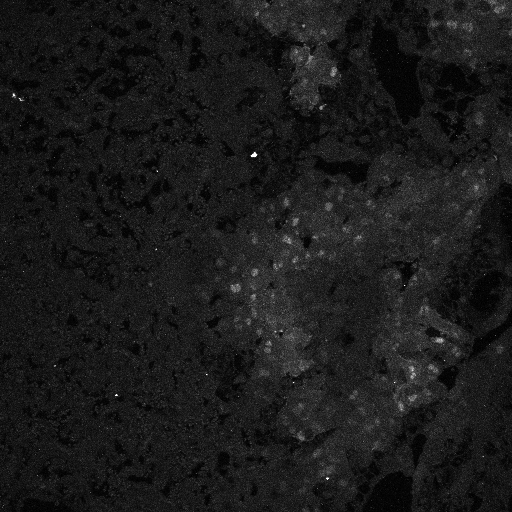

Supplement: Supplementary file 7 — Source Data Fig. 6 [file 44319_2024_97_MOESM7_ESM.zip › Figure 6/6B/Representative_Ctrl_PMDandMCT1.tif]
