# Supplementary material for: Appropriate glycemic management protects the germline but not the uterine environment in hyperglycemia
Source: EMBO Rep. 2024 Mar 15;25(4):11. doi: 10.1038/s44319-024-00097-7 (PMC11014859; doi:10.1038/s44319-024-00097-7)
Supplement: Supplementary file 9 — Expanded View Figures [file 44319_2024_97_MOESM9_ESM.pdf]

Expanded View Figures

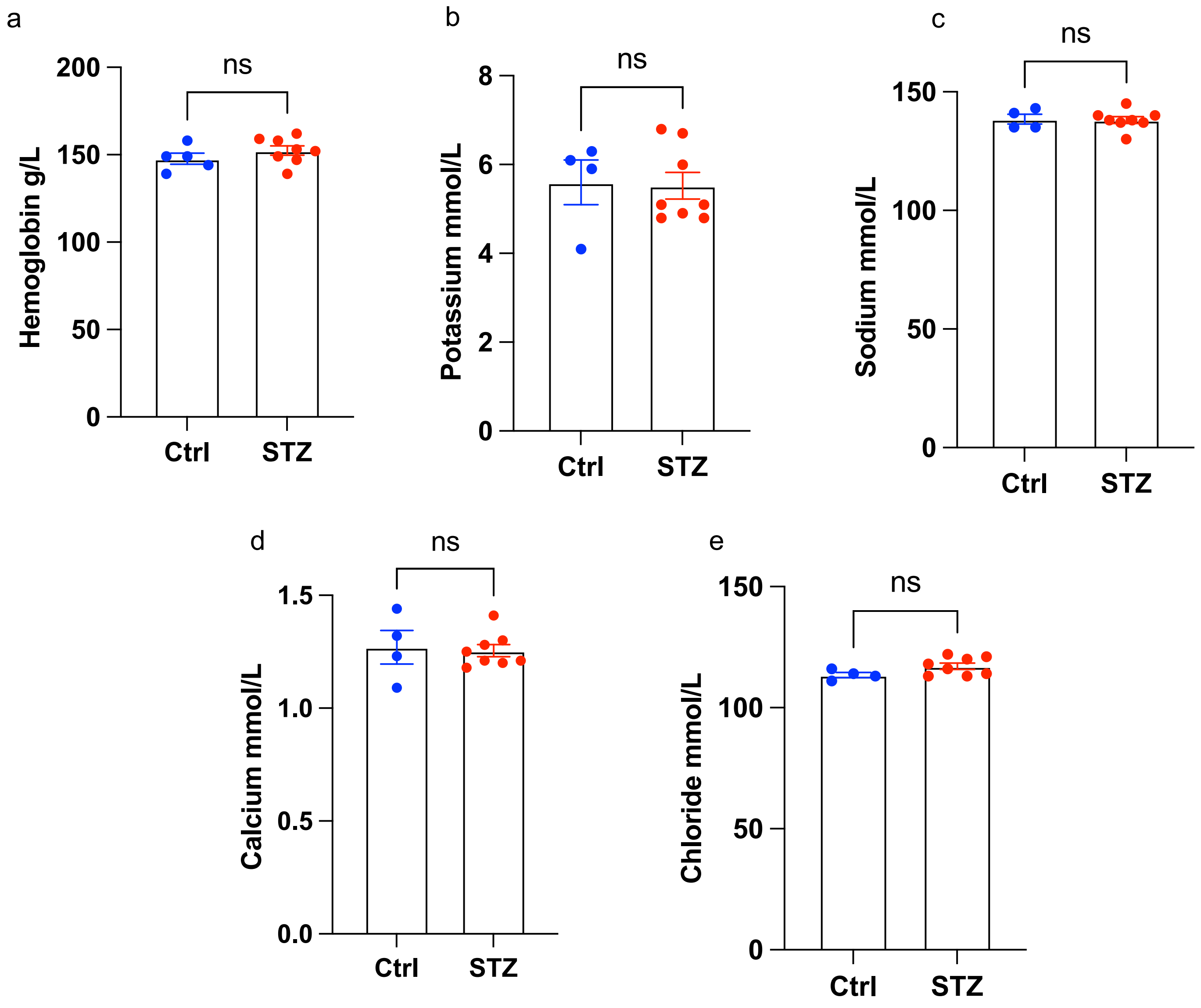

Figure EV1. Maternal mouse blood analysis.

(**A–E**) Hemoglobin ($n = 5$ control mice, $n = 8$ STZ mice) (**A**), Potassium ($n = 4$ control mice, $n = 8$ STZ mice) (**B**), Sodium ($n = 4$ control mice, $n = 8$ STZ mice) (**C**), Calcium ($n = 4$ control mice, $n = 8$ STZ mice) (**D**) and Chloride ($n = 4$ control mice, $n = 8$ STZ mice) (**E**) levels in maternal control and STZ mice. STZ, Streptozotocin; ns, non-significant. Data information: Data depicted is biological replicates. In panels (**A–E**), data is presented as mean ± SEM, and was analyzed using Student's t-test.

 © The Author(s)

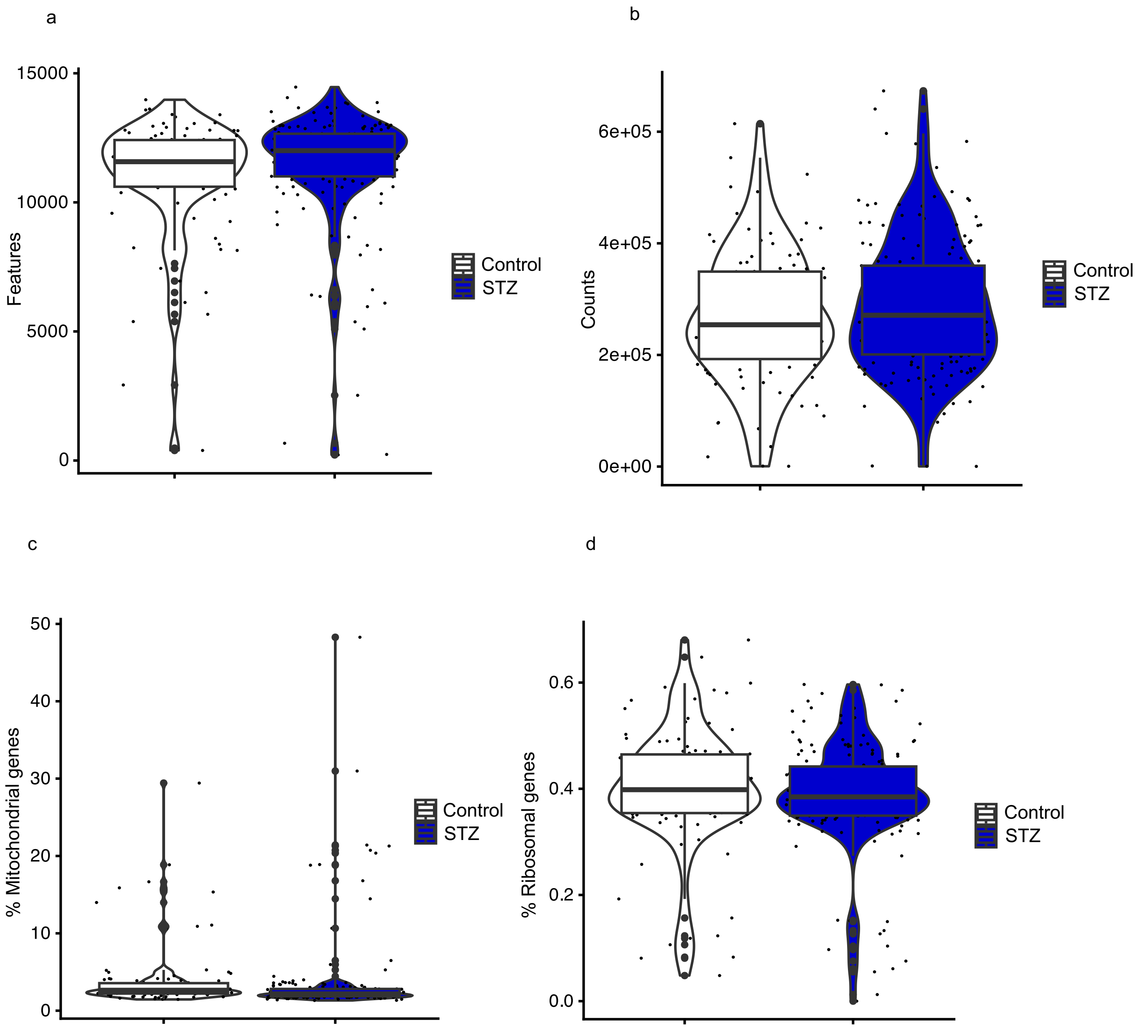

Figure EV2. QC of mouse oocyte single-cell RNAseq data.

(**A**) Amount of features (genes) per cell (5 control animals, $n = 100$ oocytes; 9 STZ animals, $n = 172$ oocytes). (**B**) Amount of counts per cell (5 control animals, $n = 100$ oocytes; 9 STZ animals, $n = 172$ oocytes). (**C**) Percentage of mitochondrial reads per cell (5 control animals, $n = 100$ oocytes; 9 STZ animals, $n = 172$ oocytes). (**D**) Amount of ribosomal reads per cell (5 control animals, $n = 100$ oocytes; 9 STZ animals, $n = 172$ oocytes). Data information: Data depicted is biological replicates and readcounts. For boxplots in panels (**A–D**), the lower and upper hinges of the box depict the 1st and 3rd quartiles, and the middle line depicts the median. The upper and lower whiskers extend to the largest or smallest value no further than 1.5 times the interquartile range (1.5*IQR) from the hinge. Data beyond the end of the whiskers are plotted as a larger dot, which are then the minimum and/or maximum values depicted. If no large dots are present, the whiskers extend to the minimum and/or maximum values.

© The Author(s)

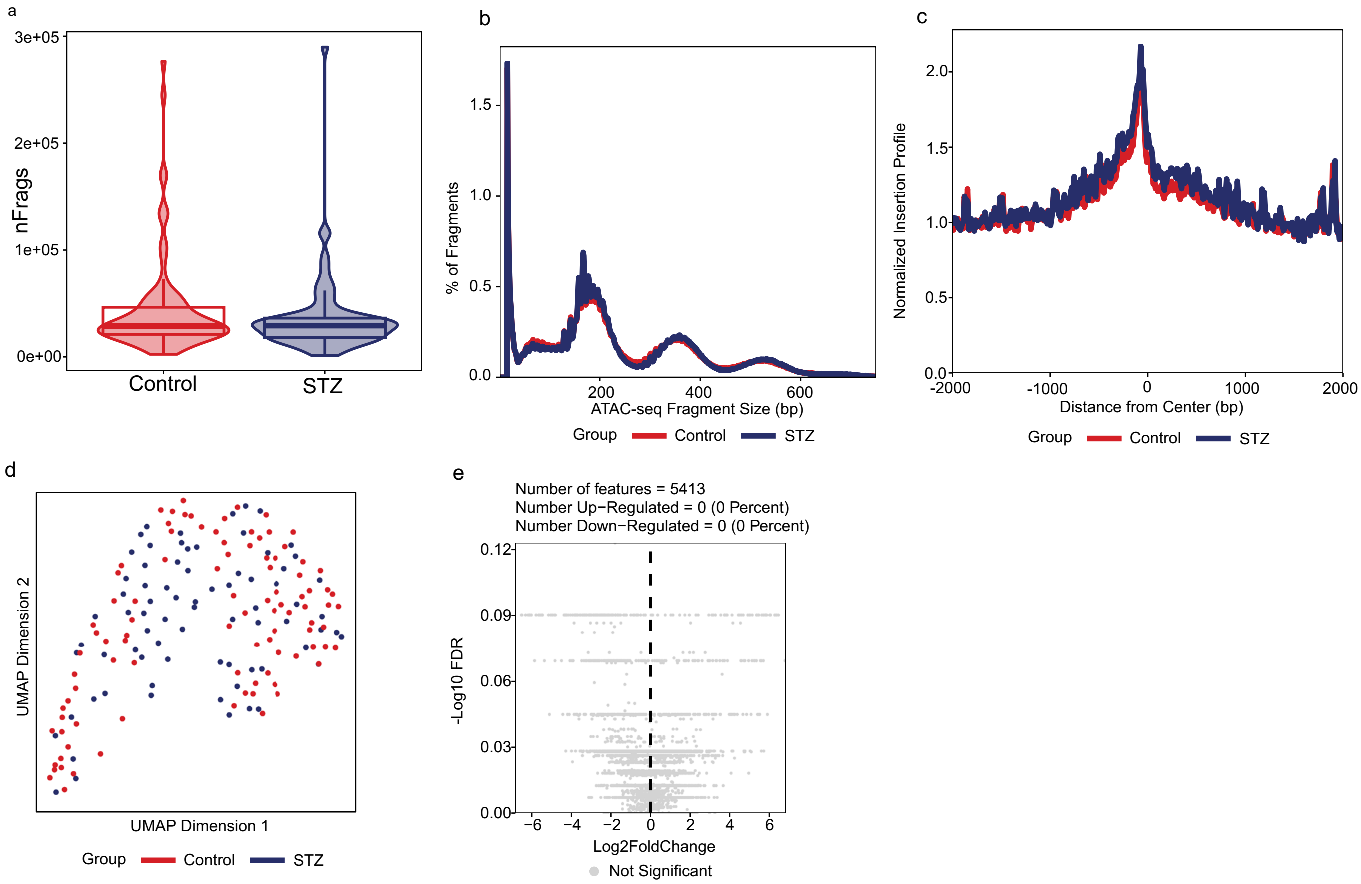

Figure EV3. Mouse oocyte single-cell ATAC seq analysis.

(**A**) Amount of unique fragments detected per cell from control ($n = 95$) and STZ oocytes ($n = 85$. (**B**) Fragment size distribution from control ($n = 95$) and STZ oocytes ($n = 85$ (**C**) TSS enrichment profile from control ($n = 95$) and STZ oocytes ($n = 85$). (**D**) UMAP depicting scATAC profiles from control ($n = 95$) and STZ oocytes ($n = 85$). (**E**) Volcano plot displaying no differentially accessible regions between control ($n = 85$) and STZ ($n = 95$) oocytes. TSS, Transcription starting site; UMAP, Uniform Manifold Approximation and Projection; STZ, Streptozotocin. Data information: Data depicted is biological replicates. For boxplots in panel (**A**), the lower and upper hinges of the box depict the 1st and 3rd quartiles, and the middle line depicts the median. The upper and lower whiskers extend to the largest or smallest value no further than 1.5 times the interquartile range (1.5*IQR) from the hinge. Data beyond the end of the whiskers are plotted as a violin plot, which are then the minimum and/or maximum values depicted.

 © The Author(s)

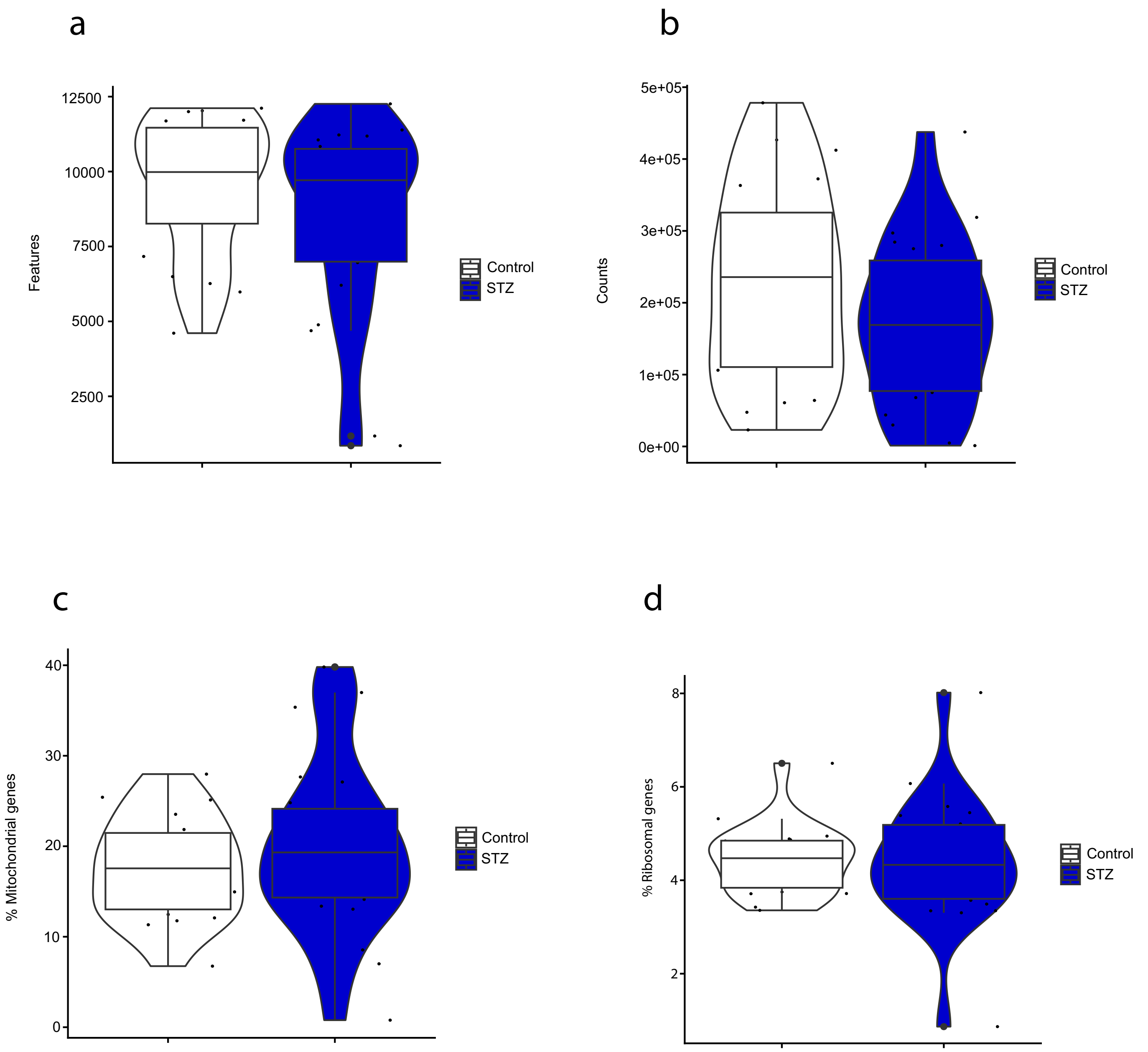

Figure EV4. QC of human oocyte single-cell RNAseq data.

(**A**) Amount of features (genes) per cell (*n* = 20 for 2.5 mM, *n* = 21 for 5 mM, *n* = 20 for 10 mM). (**B**) Amount of counts per cell (*n* = 20 for 2.5 mM, *n* = 21 for 5 mM, *n* = 20 for 10 mM). (**C**) Percentage of mitochondrial reads per cell (*n* = 20 for 2.5 mM, *n* = 21 for 5 mM, *n* = 20 for 10 mM). (**D**) Amount of ribosomal reads per cell (*n* = 20 for 2.5 mM, *n* = 21 for 5 mM, *n* = 20 for 10 mM). Data information: Data depicted is biological replicates and readcounts. For boxplots in panels (**A–D**), the lower and upper hinges of the box depict the 1st and 3rd quartiles, and the middle line depicts the median. The upper and lower whiskers extend to the largest or smallest value no further than 1.5 times the interquartile range (1.5*IQR) from the hinge. Data beyond the end of the whiskers are plotted as a larger dot, which are then the minimum and/or maximum values depicted. If no large dots are present, the whiskers extend to the minimum and/or maximum values.

© The Author(s)
